# The complete chloroplast genome of *Thalictrum baicalense* Turcz. ex Ledeb

**DOI:** 10.1080/23802359.2020.1870896

**Published:** 2021-02-11

**Authors:** Yanfeng He, Ruinan Wang, Xiangyun Gai, Pengcheng Lin, Yongxi Li, Jiuli Wang

**Affiliations:** a Key Laboratory for Tibet Plateau Phytochemistry of Qinghai Province, Qinghai Nationalities University, Xining, China; b Key Laboratory for Tibet Plateau Phytochemistry of Qinghai Province, Xining, China; c The College of Ecological Environment and Resources, Qinghai Nationalities University, Xining, China

**Keywords:** *Thalictrum baicalense*, complete chloroplast genome, phylogenetic analysis

## Abstract

*Thalictrum baicalense* Turcz. ex Ledeb. is a well-known herbaceous perennid that has been used as a traditional medicine to treat influenza, hepatitis, and detoxfeaction. In this study, we release and detail the complete chloroplast genome sequences of *T. baicalense*. The whole chloroplast genome was 155,859 bp in length and comprised 130 genes, including 84 protein-coding genes, 37 tRNA genes, eight rRNA genes. The *T. baicalense* chloroplast genome had a GC content of 38.39%. The phylogenetic relationships inferred that *T. baicalense, T. tenue*, *T. minus* and *T. petaloideum* are closely related to each other within the genus *Thalictrum*.

*Thalictrum baicalense* Turcz. ex Ledeb. (Ranunculaceae) is a herbaceous perennid distributed in China, Korea, Russia and other north warm regions (Wang et al. 2001). The dried root of *T. baicalense* have the effects of clearing heat, anti-inflammatory and antiviral. It has been used in the treatment of hepatitis, dysentery and detoxfeaction in traditional Chinese medicine. In addition, it is reported in the literature that the genus *Thalictrum* comprises about 150 species worldwide. There are 76 species throughout China, of which about 30 species have been used as traditional folk medicines to treat influenza, cancer, and conjunctivitis (Chen et al. 2003; Xue et al. 2020).

The complete chloroplast (cp) genome can provide valuable genomic information for the phylogenetics study and the conservation of rare species (Weng et al. 2014). However, the cp genome of *T. baicalense* has not been fully sequenced. In this study, we sequenced and analyzed the cp genome of *T. baicalense* based on Illumina pair-end sequencing and compared it with other genus cp genome sequences. it would not only promote the phylogenetics study in family Ranunculaceae but also provide useful genetic information for the protection of *T. baicalense* and other related species.

The samples of *T. baicalense* were collected in the Qingshashan mountain (102°01′6.42″E, 36°17′14.25″N) and the voucher specimens (He202008) are deposited in the Herbarium of College of Pharmacy, Qinghai Nationalities University, Xining, China. The total DNA of *T. baicalense* was extracted using Plant Genomic DNA Kit (DP350; TIANGEN Biotech(Beijing) Co.,Ltd.). High-throughput sequencing was performed using an Illumina NovaSeq 6000 series sequencer (PE150) by Nanjing Genepioneer Biotechnologies Inc. (Nanjing, China), and 8.04 GB of raw data was generated. The raw paired-end reads were filtered using the fastp program (Chen et al. 2018). The high-quality reads were applied to a *de novo* assembly performed using the program SPAdes assembler v3.10.1 (Bankevich et al. 2012). The assembled genome was annotated using CPGAVAS2 (Shi et al. 2019).

The complete cp genome (MW133265) of *T. baicalense* was 155,859 bp in length having 38.39% of total GC content. This cp genome has a typical quadripartite structure, containing a large single-copy region (LSC) of 85,258 bp, a small single-copy region (SSC) of 17,637 bp, and 2 inverted repeat (IR) regions of 26,482 bp. A total of 130 genes are successfully annotated, including 84 protein-codon genes, 37 tRNA genes and 8 rRNA genes. The tRNA genes are distributed throughout the whole genome with 22 in the LSC, 1 in the SSC, and 14 in the IR regions, while rRNAs are only situated in the IR regions. In IR, six protein-coding genes (*ndhB*, *rpl2*, *rpl23*, *rps7*, *rps12*, and *ycf2*), seven tRNA genes (*trnA-UGC*, *trnI-CAU*, *trnI-GAU*, *trnL-CAA*, *trnN-GUU*, *trnR-ACG*, and *trnV-GAC*), and four rRNA genes (*rrn4.5*, *rrn5*, *rrn16*, and *rrn23*) have been coded. Among the protein-coding genes, two genes (*clpP* and *ycf3*) contained two introns, and other nine genes (*atpF*, *ndhA*, *ndhB*, *petB*, *petD*, *rpl16*, *rpl2*, *rpoC1*, *rps12* and *rps16*) had one intron each.

In order to reveal the phylogenetic position of *T. baicalense* with its close relatives of Ranunculaceae, a phylogenetic analysis was performed based on 21 complete chloroplast genomes of Ranunculaceae. The complete chloroplast genomes were aligned by MAFFT v7.307 (Katoh and Standley 2013) and the maximum-likelihood tree (Figure 1) was built using MEGA7 (Kumar et al. 2016). Using the Tamura-Nei model model the ML phylogenetic analysis were conducted with MEGA v7.0.26 generating 1000 bootstrap replicates to determine measures of nodal support with each run initiating from a random starting tree. According to the result (Figure 1), *T. baicalense* has close relationship with the species *T. tenue*, *T. minus* and *T. petaloideum*.

**Figure 1.**
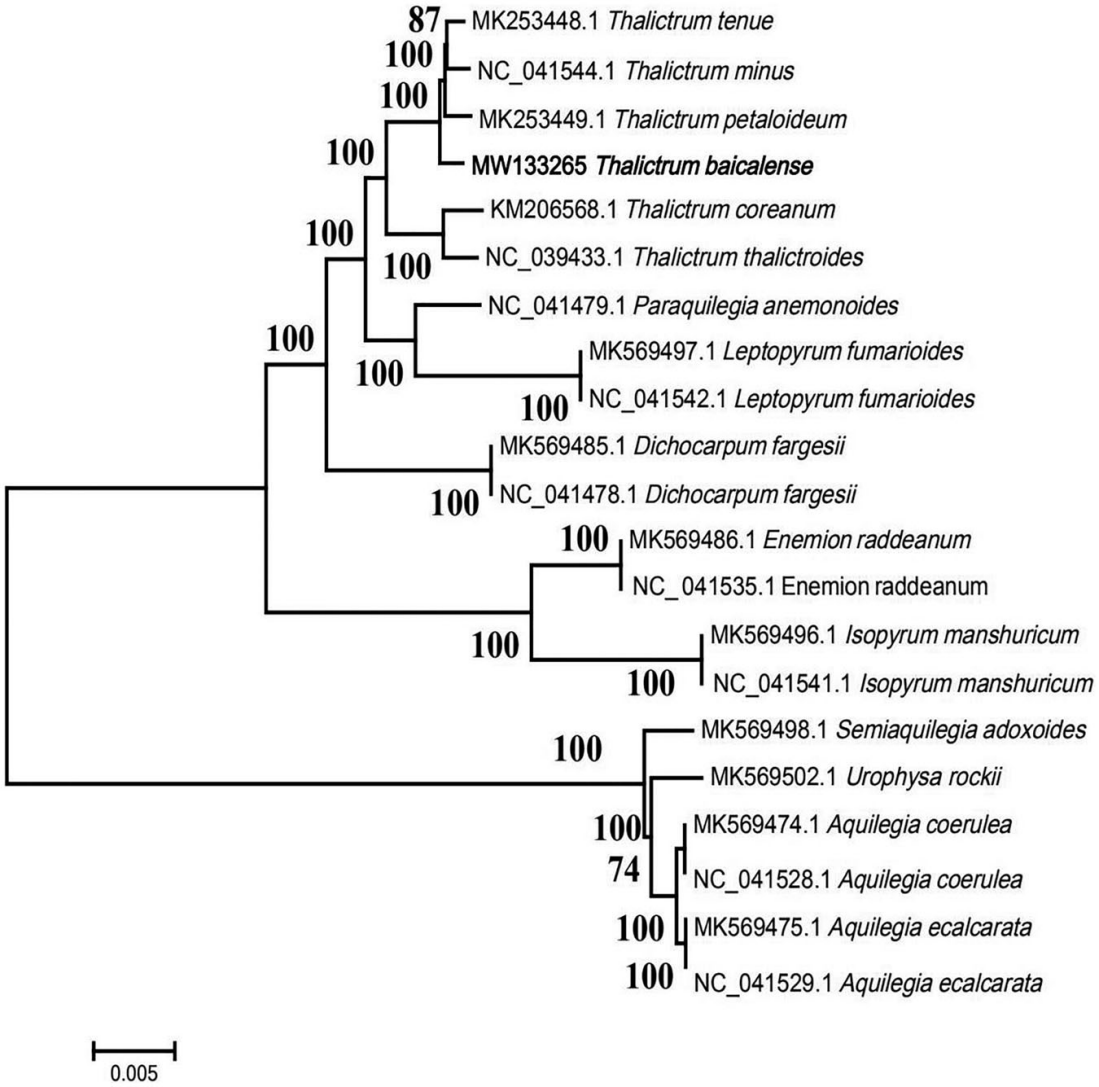
The maximum-likelihood (ML) phylogenetic tree of 21 complete chloroplast genome sequences.

## Data Availability

The data that support the results of this study are openly available in GeneBank (https://www.ncbi.nlm.nih.gov/genbank/) under the accession numbers MW133265 after the paper has been published. The associated ‘BioProject,’ ‘SRA,’ and ‘Bio-Sample’ numbers are PRJNA680221, SRR13178662, and SAMN16872741 respectively.
